# Supplementary figures and images for: Transcriptional suppression of sphingolipid catabolism controls pathogen resistance in C. elegans
Source: PLoS Pathog. 2023 Oct 31;19(10):e1011730. doi: 10.1371/journal.ppat.1011730 (PMC10637724; doi:10.1371/journal.ppat.1011730)

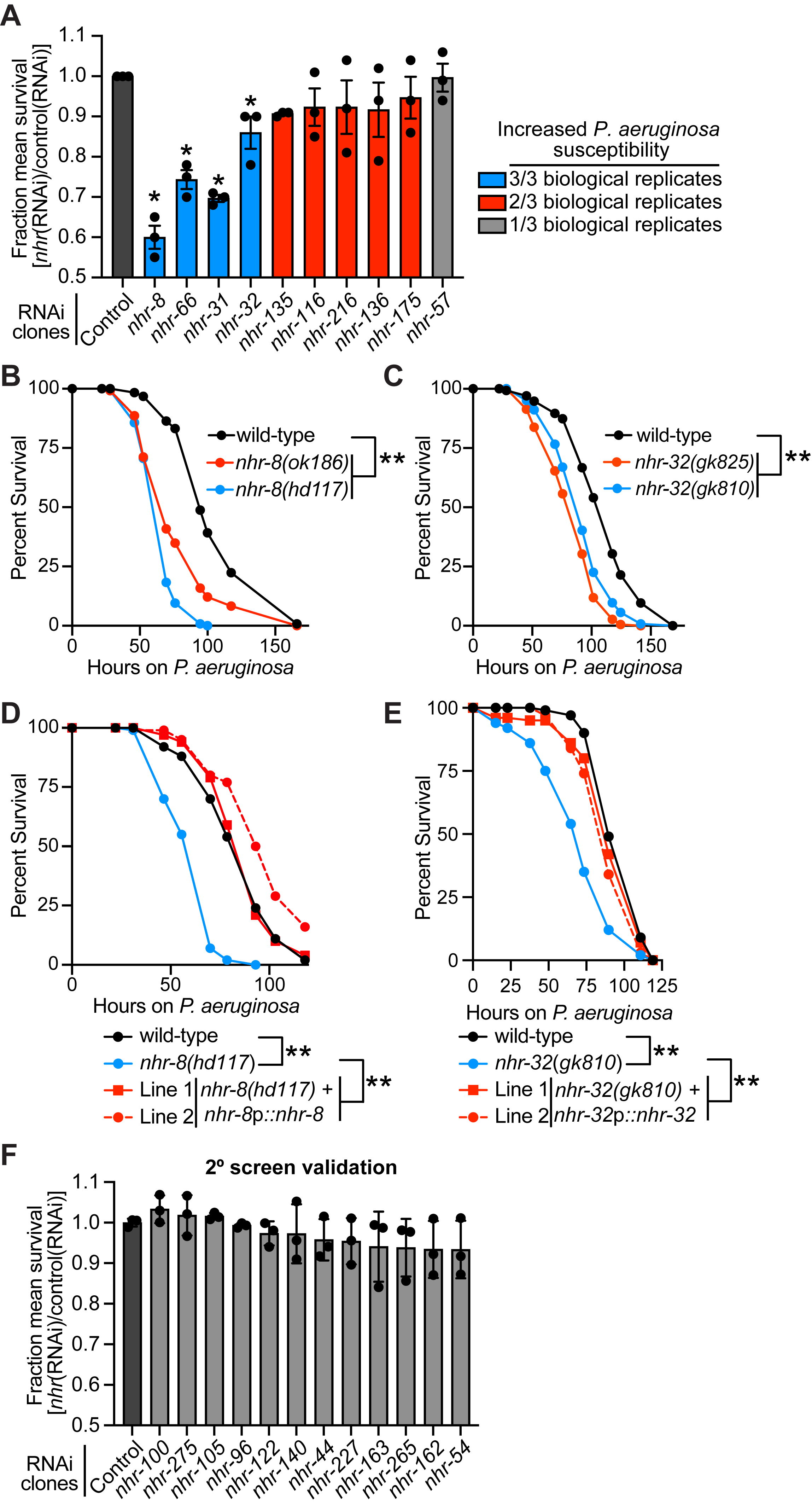

Supplement: S1 Fig — A and F. Fraction mean survival during P. aeruginosa infection of the indicated genotypes. See legend for Fig 1C. * p<0.05 (log rank) in 3 of 3 RNAi biological replicates. B-E. C. elegans-P. aeruginosa pathogenesis assays, as described for Fig 1E. **p<0.01 (log rank). Related to Fig 1. (TIF) [file ppat.1011730.s001.tif]

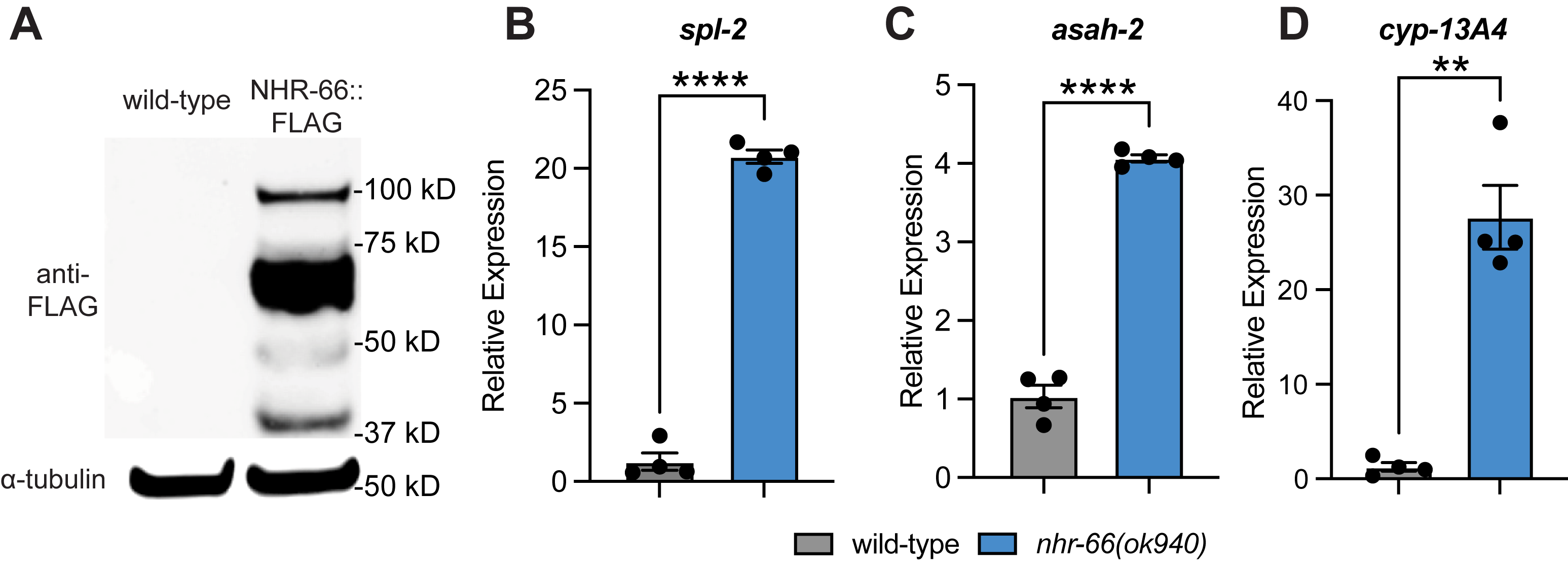

Supplement: S2 Fig — A. Western blot analysis of lysates from wild-type and NHR-66::3xFLAG animals probed with antibodies targeting the FLAG epitope (anti-FLAG) and α-tubulin (anti- tubulin). B–D. qRT-PCR analysis of the indicated genes in wild-type and nhr-66(ok940) mutant animals. Data are the average of four independent replicates with error bars representing SEM. **p<0.01, ****p<0.0001 Student’s unpaired t-test). Source data for this figure is in S6 Table. Related to Fig 2. (TIF) [file ppat.1011730.s002.tif]

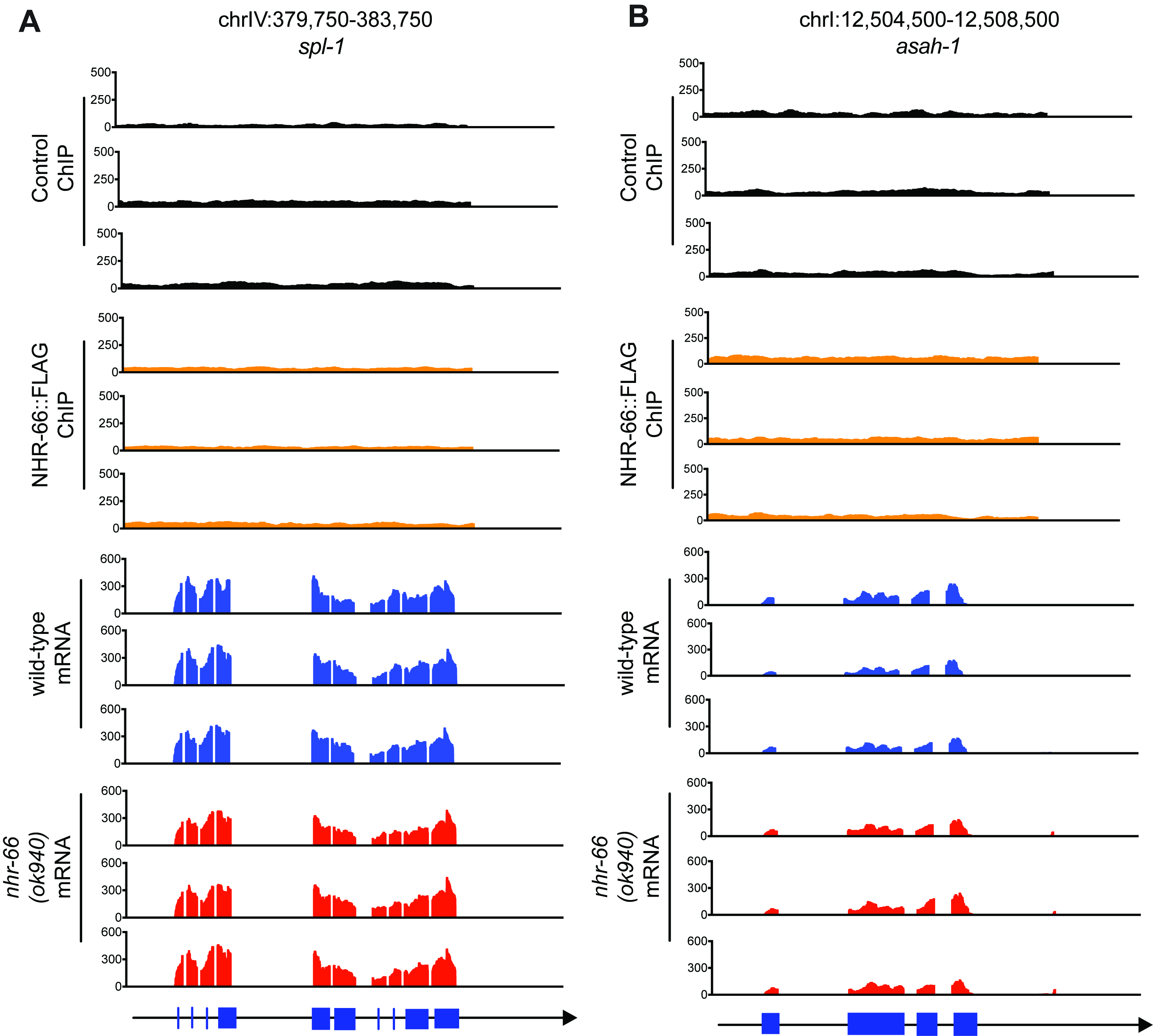

Supplement: S3 Fig — A-B. ChIP-seq and mRNA-seq profiles from each of the three biological replicates are presented for spl-1 (A), and asah-1 (B). The y-axis is the number of reads (log2). A gene model shows the location of the exons (blue) of the indicated genes. Related to Fig 3. (TIF) [file ppat.1011730.s003.tif]

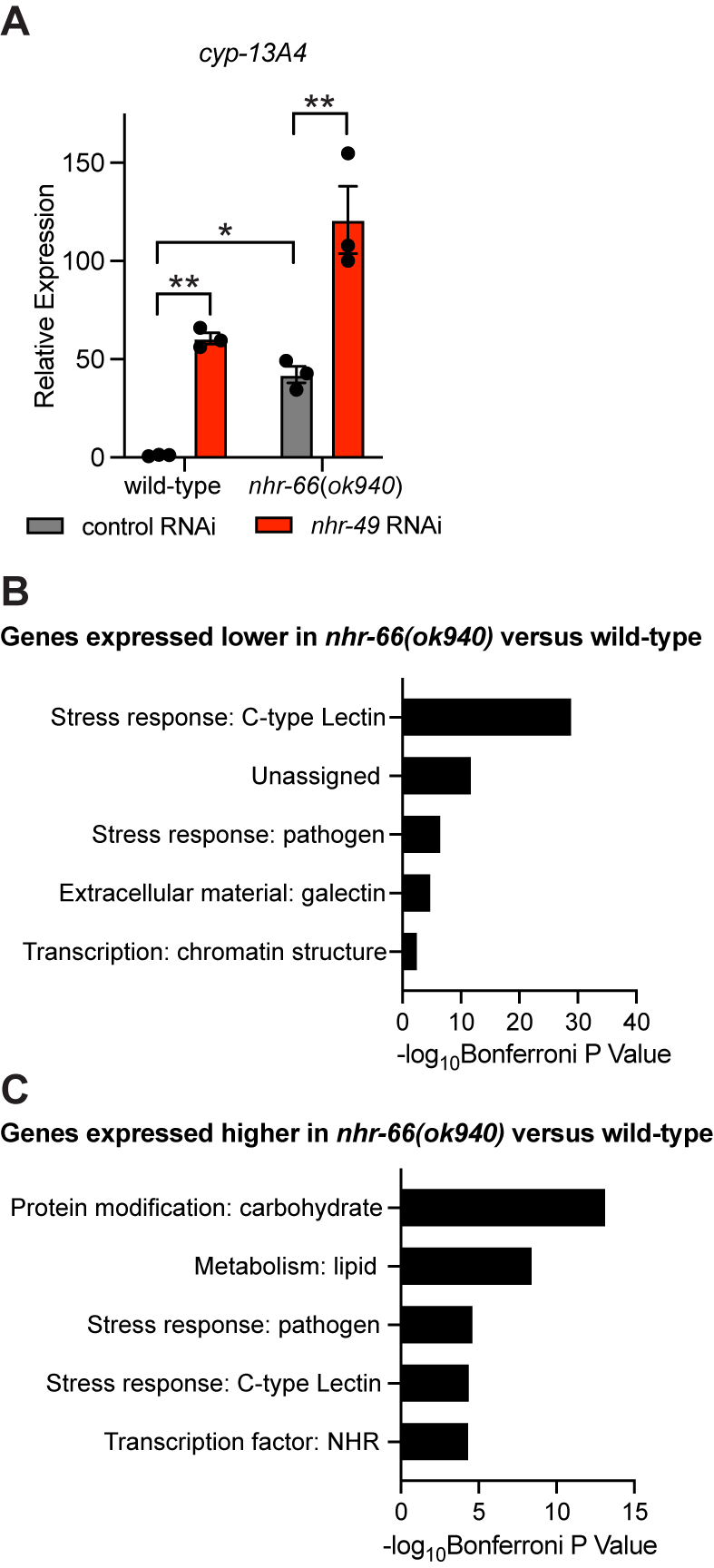

Supplement: S4 Fig — A. qRT-PCR data as described in Fig 5D. *p<0.05, ** p<0.01, *** p<0.001, **** p<0.0001 (two-way ANOVA with Tukey’s multiple comparisons test). B and C. Gene enrichment analyses for the genes whose transcription are indirectly dependent on nhr-66 is shown. The most significantly enriched categories, reported as the log10 transformation of the p-value for the enrichment of each category, are shown for genes that are expressed lower (B) and those that are expressed higher (C) in nhr-66(ok940) mutants. (TIF) [file ppat.1011730.s004.tif]
